# Chlorella diet alters mitochondrial cardiolipin contents differentially in organs of *Danio rerio* analyzed by a lipidomics approach

**DOI:** 10.1371/journal.pone.0193042

**Published:** 2018-03-01

**Authors:** Yu-Jen Chao, Wen-Hsin Wu, Maria Balazova, Ting-Yuan Wu, Jamie Lin, Yi-Wen Liu, Yuan-Hao Howard Hsu

**Affiliations:** 1 Department of Chemistry, Tunghai University, Taichung, Taiwan; 2 Institute of Animal Biochemistry and Genetics, Centre of Biosciences, Slovak Academy of Sciences, Bratislava, Slovakia; 3 Department of Life Science, Tunghai University, Taichung, Taiwan; 4 Life Science Research Center, Tunghai University, Taichung, Taiwan; Virginia Commonwealth University Department of Internal Medicine, UNITED STATES

## Abstract

The zebrafish (*Danio rerio*) is an important and widely used vertebrate model organism for the study of human diseases which include disorders caused by dysfunctional mitochondria. Mitochondria play an essential role in both energy metabolism and apoptosis, which are mediated through a mitochondrial phospholipid cardiolipin (CL). In order to examine the cardiolipin profile in the zebrafish model, we developed a CL analysis platform by using liquid chromatography-mass spectrometry (LC-MS). Meanwhile, we tested whether chlorella diet would alter the CL profile in the larval fish, and in various organs of the adult fish. The results showed that chlorella diet increased the chain length of CL in larval fish. In the adult zebrafish, the distribution patterns of CL species were similar between the adult brain and eye tissues, and between the heart and muscles. Interestingly, monolyso-cardiolipin (MLCL) was not detected in brain and eyes but found in other examined tissues, indicating a different remodeling mechanism to maintain the CL integrity. While the adult zebrafish were fed with chlorella for four weeks, the CL distribution showed an increase of the species of saturated acyl chains in the brain and eyes, but a decrease in the other organs. Moreover, chlorella diet led to a decrease of MLCL percentage in organs except the non-MLCL-containing brain and eyes. The CL analysis in the zebrafish provides an important tool for studying the mechanism of mitochondria diseases, and may also be useful for testing medical regimens targeting against the Barth Syndrome.

## Introduction

Zebrafish is an established vertebrate model for biomedical research due to its embryo transparency, low-cost maintenance, high similarity to human genetics, and feasibility for drug-screening. Most organs in zebrafish, except those of the reproduction system, start to function before 5 days post fertilization, which facilitates phenotypic manifestation of genetic disorders early in development. The zebrafish disease models of anemia, leukemia, cancer, heart disorder, muscle disorder and human degenerative diseases have been created [1, 2]. It is noteworthy that the zebrafish has emerged as a model for mitochondrial biology and diseases. The mitochondria were shown to be abundantly distributed at multiple tissues such as the eye, brain, heart and muscle in the developing zebrafish. Human mitochondrial diseases that have been replicated in the zebrafish larvae include those affecting the cardiovascular, nervous, visual, muscular and hematopoietic systems [3–8]. Zebrafish also gains its popularity in the lipid research to tackle the diseases caused by lipid metabolism abnormality [9]. Supplementation of polyunsaturated fatty acids (PUFA) to the zebrafish and the trout can change the phospholipid composition in mitochondria and affect the mitochondrial activity to produce ATP [10, 11].

Chlorella is a popular nutritional supplementation containing more than 60% of PUFA in the total fatty acids content [12]. Active components from the chlorella extracts have been shown to have anticancer activity, [13–15] and anti-inflammation effects [16–18]. The supplemented PUFA can be incorporated into the mitochondrial phospholipids cardiolipin (CL) and alter the fatty acyl chain compositions of CL in H9c2 cells [19]. CL content in mitochondria can fluctuate along with the environmental fatty acid content. Starvation and replenishment of serum have been shown to affect the CL compositions in cancer cell lines [20]. Therefore, supplementation of fatty acids can affect the inner membrane structure of mitochondria and ATP synthesis efficiency though the incorporation of fatty acid into mitochondrial CL.

Mitochondrial activity can be evaluated by quantification of the proton circuits, mitochondrial or cell respiratory control, membrane potential and adenine nucleotides, which provide different perspectives of the mitochondria [21]. CL is a major membrane component critical for the curvature of crista of inner mitochondrial membrane [22, 23]. Mitochondrial dysfunction is related to the errors in quality and quantity of CL compositions, which decrease the efficiency of energy processing. CL(s) containing multiple PUFA are particularly susceptible to ROS-induced oxidation, which causes the inactivation of electron transport complex I, III and IV [24–26]. The correct CL/monolyso-cardiolipin (MLCL) ratio is directly related to the structure and function of mitochondria [27]. Mass spectrometry has proved to be a useful method for the quantification of CL(s) and the determination of CL/ MLCL [28].

The zebrafish has been utilized as a model for studying human Barth syndrome which is caused by the mutation of the CL remodeling enzyme Tafazzin [4, 29]. Moreover, novel hematopoietic function of CL remodeling enzyme Lysocardiolipin Acyltransferase has been discovered based on a zebrafish mutant study [30, 31]. However, the methodology of profiling CL as well as its remodeling in the zebrafish has remain unestablished. In this study, we used the mass spectrometry (MS) approach to demonstrate that the CL profile of the larval zebrafish can be remodeled upon the dietary addition of high-PUFA-containing chlorella. Moreover, the CL and CL/ MLCL profiles for various adult organs have been determined. We showed that brain and eye tissues contained similar CL and CL/ MLCL profiles, which were distinct from those of heart, muscle, liver and ovary. In summary, we have established a lipodomics method to detect the CL remodeling in both larval and adult zebrafish.

## Materials and methods

### Ethics statement

All of the zebrafish-use protocols in this research were reviewed and approved by the Institutional Animal Care and Use Committee of Tunghai University (IRB Approval NO. 105–28).

### Materials

Larval AP100 (<100 microns, Zeigler, U.S.A) was used as standard larval food. NovoBel tropical flakes for aquarium fish (JBL, Germany) was ground and used as standard adult fish food. The chlorella was purchased from D.Y.BIO, Taiwan. Tetramyristoyl cardiolipin standard CL(14:0)_4_ was purchased from Avanti Polar Lipids, USA. *Siganus* fish was purchased from local fish market. The pig liver was purchased from local market. Fish oil 1000 mg in softgels was purchased from Kirkland Signature of Costco, US. Thin-layer chromatography (TLC) plate was purchased from Merck, Germany.

### Zebrafish lines and maintenance

Zebrafish (*Danio rerio*) hatched from the same batch of eggs were maintained following standard protocols [32]. The chlorella was ground before chlorella supplementation. The chlorella plus fish oil-supplemented food was prepared by mixing 1 g of chlorella and 0.071 g of fish oil. Nine 15-months old zebrafish were divided into 3 groups, and maintained with designated diets: standard adult fish food, chlorella, and fish oil-supplemented chlorella; for four weeks. Because the experiment of one adult fish in the chlorella treatment failed during lipid extraction process, the mass spectrometry data of six organs from this group were duplicated from two fish. All fish were anaesthetized on ice and weighed, and the adult fish were dissected to collect tissues for weighing and storage at -20 °C prior to experiments. Nine 1-week old larvae were also separated into 3 groups and fed with the designated diet: standard larval fish food, chlorella and fish oil-supplemented chlorella for two weeks before harvest. The fresh *Siganus* fish from the Taiwan Strait was frozen in -20 °C and dissected to acquire organs within 24 hr. after thawing.

### Fatty acid profiling

To analyze the fatty acid compositions of chlorella and fish food, 0.2 g of chlorella or 1 g of fish food was grounded and placed in a glass tube. The samples were added 0.5 ml of 3 M KOH and 2.5 ml MeOH and then sonicated for 30 sec. After adding additional 2 ml of MeOH to maintain the KOH:MeOH = 1:9, the samples were vortexed and heated in water bath at 80 °C for one hour. After the samples cooled down to room temperature, the samples were adjusted to pH 7. The fatty acids were initially extracted by Bligh-Dyer’s method and then added 2 ml of hexane for further extraction. The extraction of fatty acids from fish oil was performed according to the same method without Bligh-Dyer’s extraction. After the extraction, the hexane was dried under N_2_ gas, and the fatty acids were resuspended by ACN:IPA:DDW = 65:30:5 for mass spectrometry analysis.

### Lipid extraction

The total lipids in the collected tissues from zebrafish were extracted by the Bligh-Dyer’s method [33]. Before extraction, tissues were homogenized by 2-ml Dounce tissue grinder in 1 ml methanol. The internal standard, 125 ng tetramyristoyl cardiolipin CL(14:0)_4_, was then added to the tissue sample. The homogenized tissue was collected, and the residual tissue on the grinder was washed off with 1 ml MeOH. A total of 2 ml tissue sample in MeOH was transferred to a glass tube. After pulse sonication by 80W UP-80 ultrasonic processor (CT ChromTech, Taiwan) for 2 min on ice, 1 mL of dichloromethane was added to samples and vortexed for 10 min. Then 1 ml of dichloromethane and 1 ml of distilled deionized water were added to samples and vortexed for 10 min. The lower phase in the glass tube was collected after centrifugation at 3000 rpm for 5 min.

### Thin-layer chromatography

TLC was performed on a 20 cm × 20 cm sheet coated with thin layer of silica gel. The cellulose paper was placed into the closed TLC chamber with 98 ml of mobile phase (CHCl_3_:MeOH:HAc = 65:25:8) for one hour. The lipid extracts from the organs were dissolved in 200 μl of chloroform and MeOH in 2:1 ratio. The concentration of the Pi in each samples were determined by the phosphate quantification test and UV absorbance spectrometry at 830 nm. A total of 8 μg phosphorous of the lipid extracts were spotted onto the TLC plates. On the spotted plate, the heart and muscle samples contain only 1.15 μg and 1.8 μg of phosphorous, respectively. The TLC plates were then placed vertically in the TLC chamber for 2 hours, and then dried and developed in iodine vapor for 5 min.

### Phosphate quantification

Each phospholipid spot on the TLC plate was scratched down and put into a glass tube. After adding 200 μl of H_2_SO_4_:HClO_4_ = 9:1, the samples were incubated at 200 °C for 30 min. The samples were allowed to cool down for 10 min at room temperature and added 4.8 ml of solution, containing 500 ml of 0.26% (NH_4_)_6_Mo_7_O_24_. 4H_2_O: 22 ml of ANSA. ANSA stock solution is composed of 16 g K_2_S_2_O_5_, 0.252 g C_10_H_9_NO_4_S and 0.5 g Na_2_SO_3_ in 100 ml. The samples were incubated at 105 °C for 30 min, cooled down and then gently centrifuged at 500×g for 2 min. The Pi of the phospholipids that reacts with ammonium heptamolybdate can form molybdenum blue, which was then detected by absorbance spectrometry at 830 nm.

### Gene expression evaluated by real-time quantitative PCR

Zebrafish organs were added 1 ml of TRIZOL reagent, homogenized and then sonicated on ice for 1 min. The samples were added 200 μl of chloroform for phase separation, and 500 μl of upper phase was collected to mix with 500 μl isopropanol. The mRNA was purified from the collected precipitate. The cDNA synthesis Kit from Bio-Rad was utilized for the mRNA reverse transcription. The quantitative PCR mix contained 50 ng of cDNA, 10 pmole of forward primer, 10 pmole of reverse primer and 10 μl of iQ^™^ SYBR Green Supermix (Bio-Rad) in a 20 μl reaction. The primers of tafazzin (NM_001001814.1) are forward 5’-CCT CGA GTA GGA CAG CGG AT-3’ and reverse 5’-GCA TTT CCG TCG GAT TCG TG.-3’ The primers of phospholipase A_2_ (group VI (pla2g6), NM_213097.2) are forward 5’-AAA GCC CTG ATG GTG TTT GG-3’ and reverse 5’-CAG CGT TCG ACA CCT ACA CTA-3’. The primers of GAPDH (NM_001115114.1) are forward 5’-GCA ACA CAG AAG ACC GTT GA-3’ and reverse 5’-GCC ATC AGG TCA CAT ACA CG-3’. Real-time quantitative PCR was performed on a MiniOpticon Real-Time PCR System (Bio-Rad, Hercules, CA). The cycling conditions were 180 sec at 95°C for initial polymerase activation and 40 cycles of 15 sec at 95°C and 90 sec at 60°C.

### MS analysis

The extracted total lipids from zebrafish’s tissues were dried under nitrogen gas and re-dissolved in 400 μl of acetonitrile/2-propanol/H_2_O (65:30:5) immediately. The samples were analyzed by LC/MS Ion-Trap (Bruker Corporation). A total of 50 μl dissolved sample was injected through the autosampler. HPLC mobile phases contained solution A: ACN:H_2_O (60:40), 10 mM ammonium formate, 0.1% formic acid and solution B: IPA:ACN (90:10), 10 mM ammonium formate, 0.1% formic acid[34]. Gradient was from 60% solution A to 100% solution B in 25 min and maintained 100% solution B until 45 min in an Acclaim RSLC 120 C18 2.1 mm x 100 mm 2.2 μm column (Thermo) at a flow rate of 0.2 mL/min at 55 °C. Data were further analyzed by Bruker DataAnalysis (ver.4.1). The extract ion current (XIC) of each cardiolipin species was quantitated by their relativity of XIC to internal standard. Standard curve of XIC detector response versus content of cardiolipin standard is provided in S1 Fig.

## Results

### The CL profile of the larval zebrafish is altered by a chlorella diet

**Fig 1:**
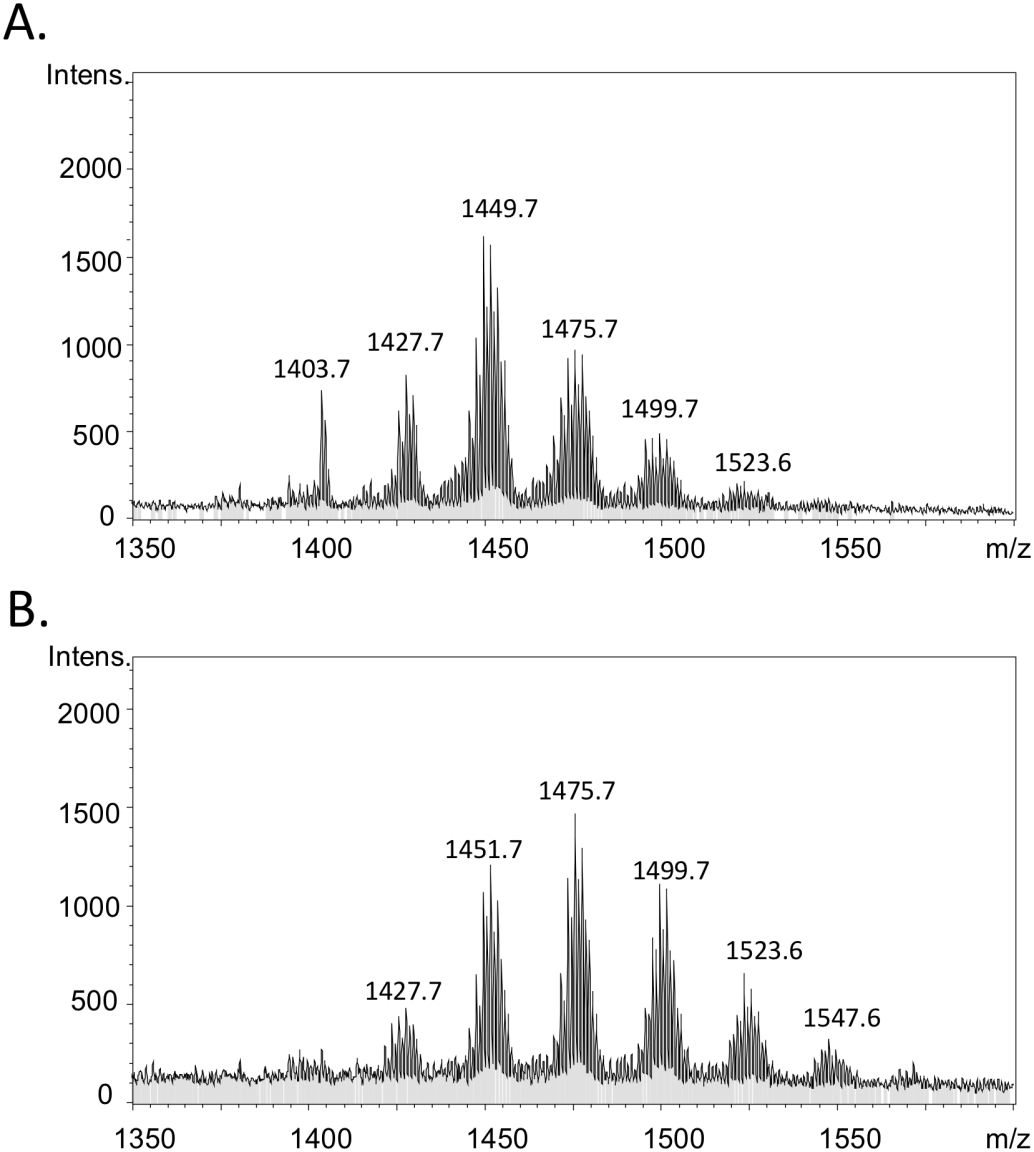
Mass spectrum of cardiolipins in the larval zebrafish. Larval fish (one-week old) was maintained with normal diet (A) and chlorella diet (B) for two weeks. After total lipid extraction, the cardiolipin was analyzed by LC-MS.

**Fig 2:**
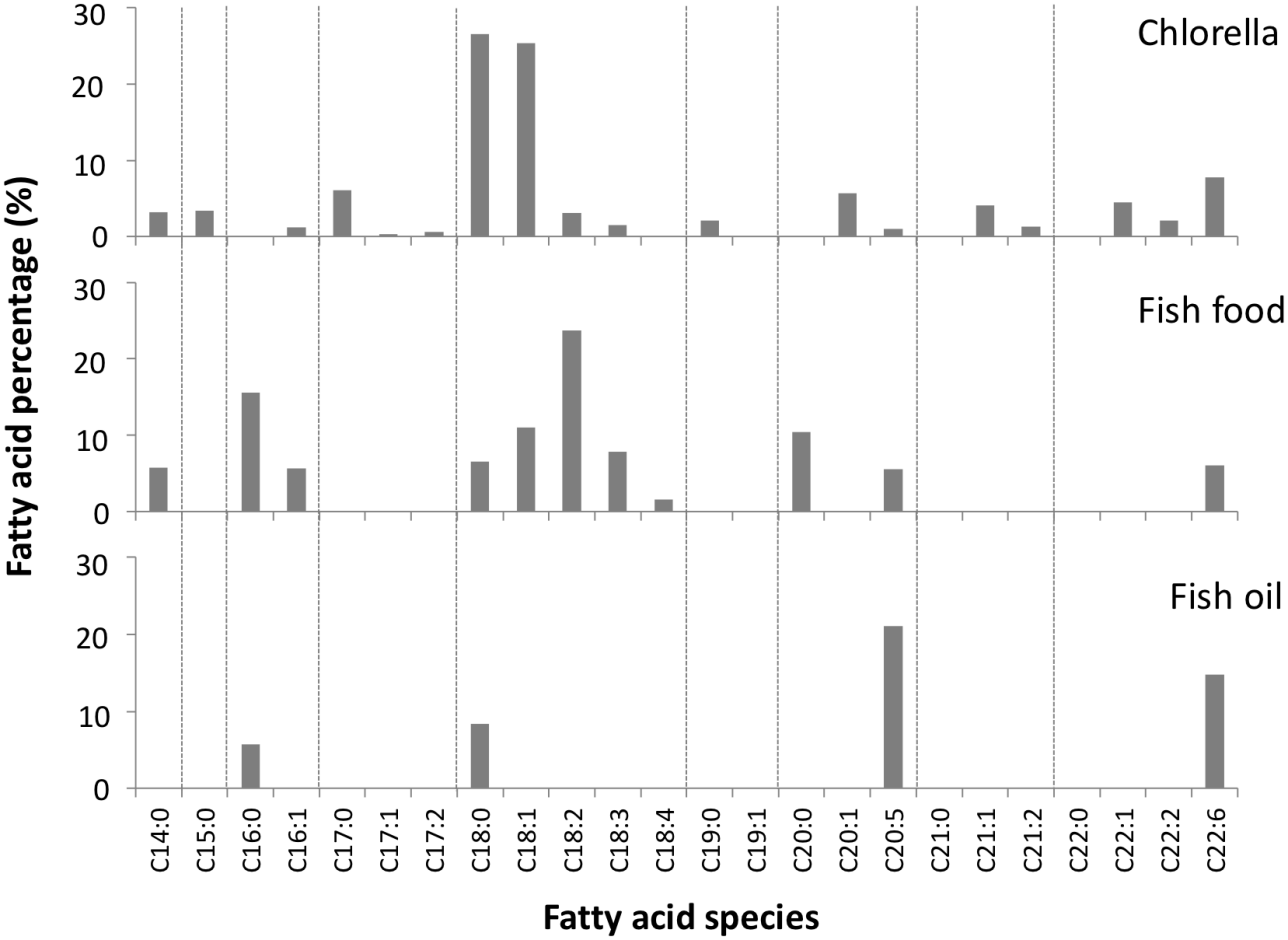
Fatty acid compositions of fish food and supplements. The fish food, chlorella and fish oil were saponified by 1M NaOH. The fatty acids were extracted by the Bligh-Dyer method and analyzed by LC-MS.

To examine the CL contents in the larval zebrafish, and to test whether the CL contents in the larval fish could be changed by the chlorella diet, 9 newly-hatched fish were collected and separated into three groups for feeding starting at the stage of 1-week post fertilization. The three groups were fed with standard larval food, chlorella, and chlorella supplemented with fish oil, respectively, for two weeks. Total lipids in the 3-week old larval fish were extracted by Bligh-Dyer’s extraction method and analyzed by LC-MS. The CL profile in larval fish fed with standard larval diet contains multiple groups of CL species, and the CL groups display normal distribution on the mass spectrometry spectrum (Fig 1). Based on the mass of the molecule, we were able to calculate the chain length and double bond number of CL. The C72 group shows the strongest intensity with 37.8%, and the CL 1449.7 m/z is the main species under standard larval diet. In the chlorella diet, we found that the main CL group shifts to C74 group, and the species 1475.7 m/z becomes the dominant CL species with 28.7%. C72 shows a major 9.7% decrease, and C76 and C78 show 5.8% and 3.6% increases of CL respectively. In the larval fish fed with chlorella, the fatty acyl chains in CL(s) shift towards higher chain length, containing more unsaturated species, suggesting that the PUFA in the chlorella diet have been incorporated into mitochondrial CL and are involved in the CL remolding. The fish oil-supplemented chlorella group did not show significant differences as compared to the chlorella-fed fish group (S2 Fig). The fatty acid compositions were different among the fish food, the chlorella and the fish oil. (Fig 2). The fish oil is mainly composed of EPA and DHA. The fish food contains 16-carbon and 18-carbon fatty acids, with lower amounts of EPA and DHA. Chlorella contains high percentage of 18:0 and 18:1 fatty acids, and versatile long chain fatty acids. This indicates that the long chain fatty acids in versatile lipid forms can be an important factor to change the CL profile in fish.

### CL profiling in the adult zebrafish

**Fig 3:**
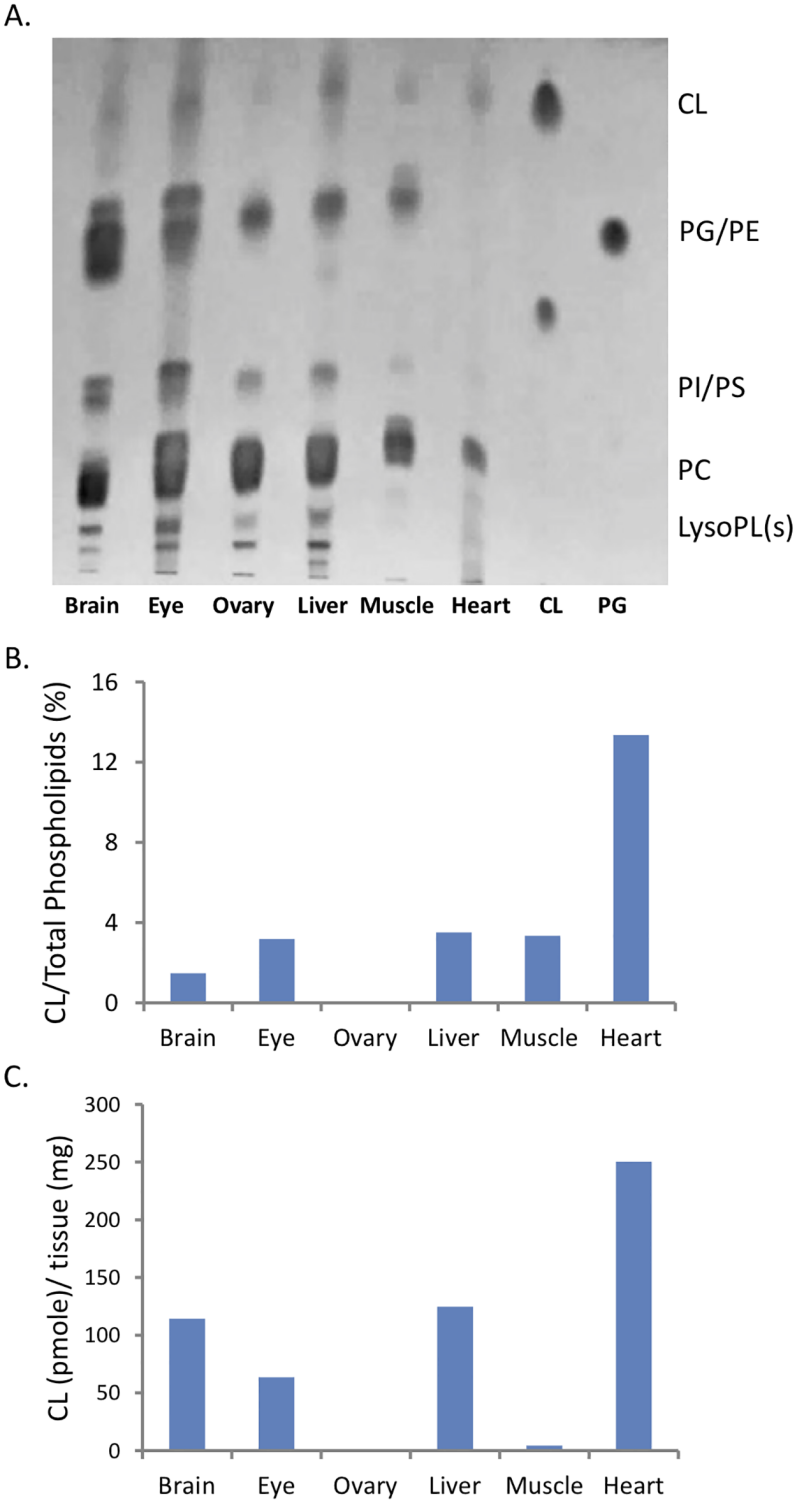
Separation and quantification of zebrafish phospholipids on TLC. (A) The phospholipids in six organs of zebra fish were separated on a TLC plate. The phospholipids equivalent to 8 μg of Pi from the brain, eye, ovary and liver were loaded into the first four lanes; and those equivalent to 1.8 and 1.15 μg of Pi from the muscle and heart samples, respectively, were loaded into next two lanes. The last two lanes were the phospholipid standards, CL and PG. (B) The ratios of the CL to total phospholipids were calculated based on the quantitated phosphate level on the TLC spots. (C) The quantities of CL in six organs were calculated based on ratios of CL on TLC plate.

Three zebrafish were sacrificed and the organs of the fish were collected. Phosphate concentration in the lipid extract of each organ was determined by the phosphate test. Among these tissues, brain and liver tissues are relatively soft, which may increase the efficiency of lipid extraction. The brain and the ovary contained more phospholipids, and incomplete homogenization of the muscle tissue led to low yield of the lipid extraction. To estimate the quantity of phospholipids, the lipid extracts were spotted onto the TLC plate for the separation of phospholipids including CL, phosphatidylglycerol (PG)/phosphatidylethanolamine (PE)/MLCL, (phosphatidylinositol) PI/phosphatidylserine (PS) and phosphatidylcholine (PC). (Fig 3A). PC was the main phospholipids in all tissues. CL was quite hydrophobic and traveled faster than other phospholipids, and its quantity was very low in the ovary. It is noteworthy that CL and PC were the most abundant phospholipids present in the heart tissue. We further quantitated all of the separated phospholipids on the TLC plate (Fig 3B). Heart tissue contains CL in 13% of total phospholipids and 250 pmole/mg tissue, indicating high amount of mitochondria in the heart (Fig 3C). All other tissues contain CL in less than 4% of total phospholipids.

**Fig 4:**
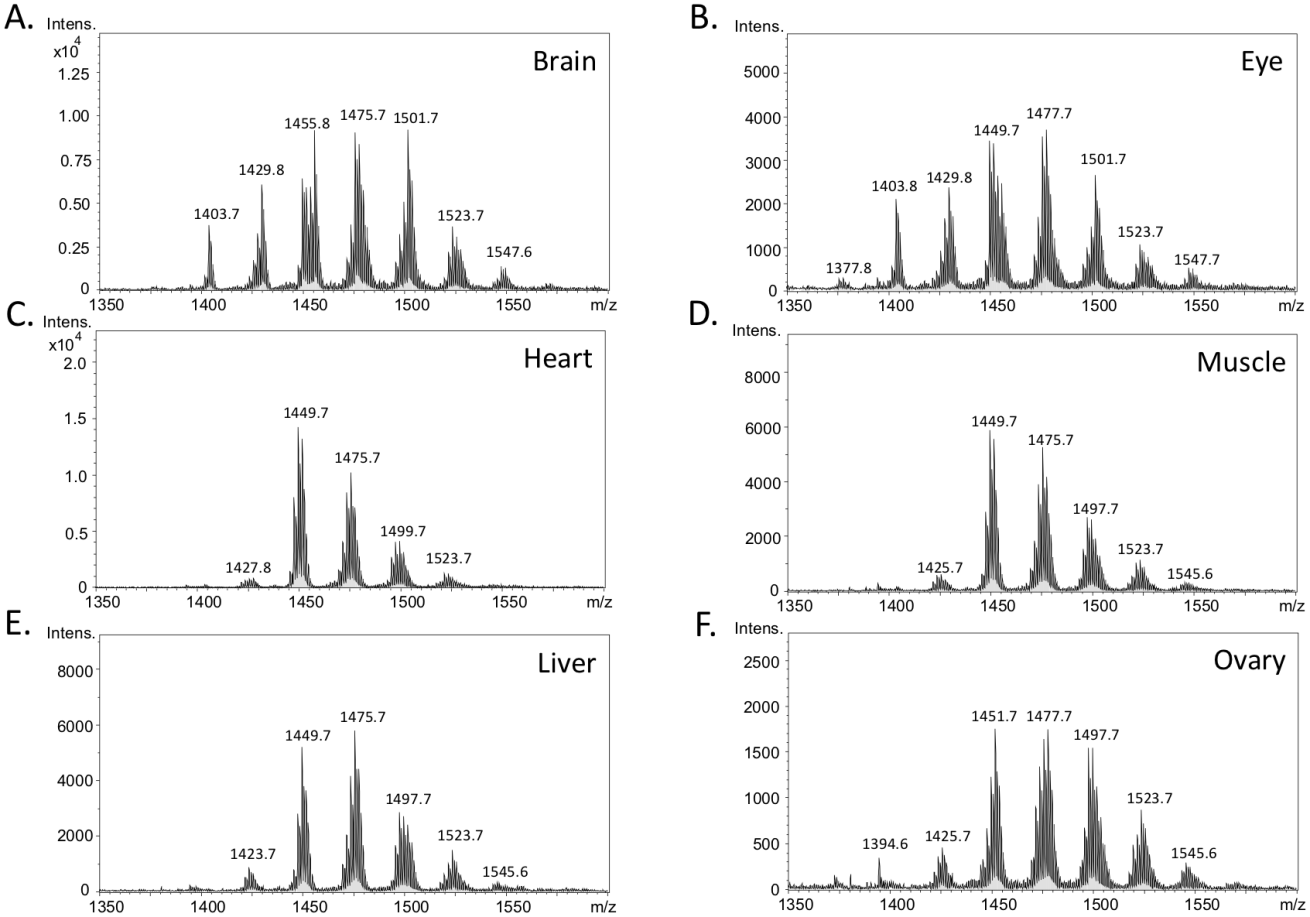
Mass spectrum of cardiolipins in different organs of the adult zebrafish. Adult female fish (fifteen-months old) was maintained with normal diet for four weeks. The lipids in various organs, including those of (A) brain, (B) eye, (C) heart, (D) muscle, (E) liver and (F) ovary, were extracted and cardiolipins were analyzed by LC-MS.

In mammals, CL has been shown to remodel differently in various cellular or animal models. In the MS analysis, detected CL species can be normally distributed as we discovered in the larvae zebrafish. Alternatively, CL can be restricted to one or two dominant symmetrical species, such as the CL found in pig liver (S3A Fig). The dominant m/z = 1448 in the mass spectrum of pig liver belongs to a symmetrical CL (18:2)_4_ species. To confirm that the CL profile of zebrafish larvae was not due to the feeding diet, we also analyzed the CL profile of the *Siganus* fish directly from ocean (S3B Fig). The CL species of *Siganus* muscle were distributed into 6 to 7 groups, similar to those detected in the larval zebrafish. Profiling the pattern of CL distribution in different organs of zebrafish, a model for human mitochondrial diseases, can benefit the analysis of effects of drug or nutrition supplementation. However, the distribution profiles of CL in adult zebrafish organs have not been reported. We hypothesized that the CL profile in different organs of zebrafish are disparate. To evaluate this hypothesis, the fish were maintained to fifteen-months old and kept with standard adult food for four weeks. The fish was sacrificed to acquire the brain, eyes, heart, muscle, liver and ovary tissues. The total lipids in each organ were extracted and analyzed by MS (Fig 4). The results displayed 7 major groups of CL in the brain and eyes. Among them, the major C72, C74 and C76 groups accounted for 24.5%, 25.6% and 20.9% of the total CL in the brain; and 24.4%, 24.3% and 17.3% in eyes. Compared to the CL profile in the brain and eyes, the CL profile in the heart and the muscle were more concentrated on C72 and C74 groups. C72 and C74 groups accounted for 37.7% and 30.4% of the total CL in the heart; and for 32.0% and 32.6% in the muscle. The CL species in these two organs shifted to lower mass on the spectrum, indicating the substitution of more unsaturated CL. Our results showed similar distribution patterns between the brain and eyes, and between the muscle and the heart, suggesting similar mitochondrial function and activities in these two groups of organs. In the liver, C72, C74 and C76 groups accounted for 26.4%, 30.9% and 19.6% of total CL, and for 22.2%, 28.4% and 22.5% in the ovary.

### Quantification of CL species in the organs of adult zebrafish

**Fig 5:**
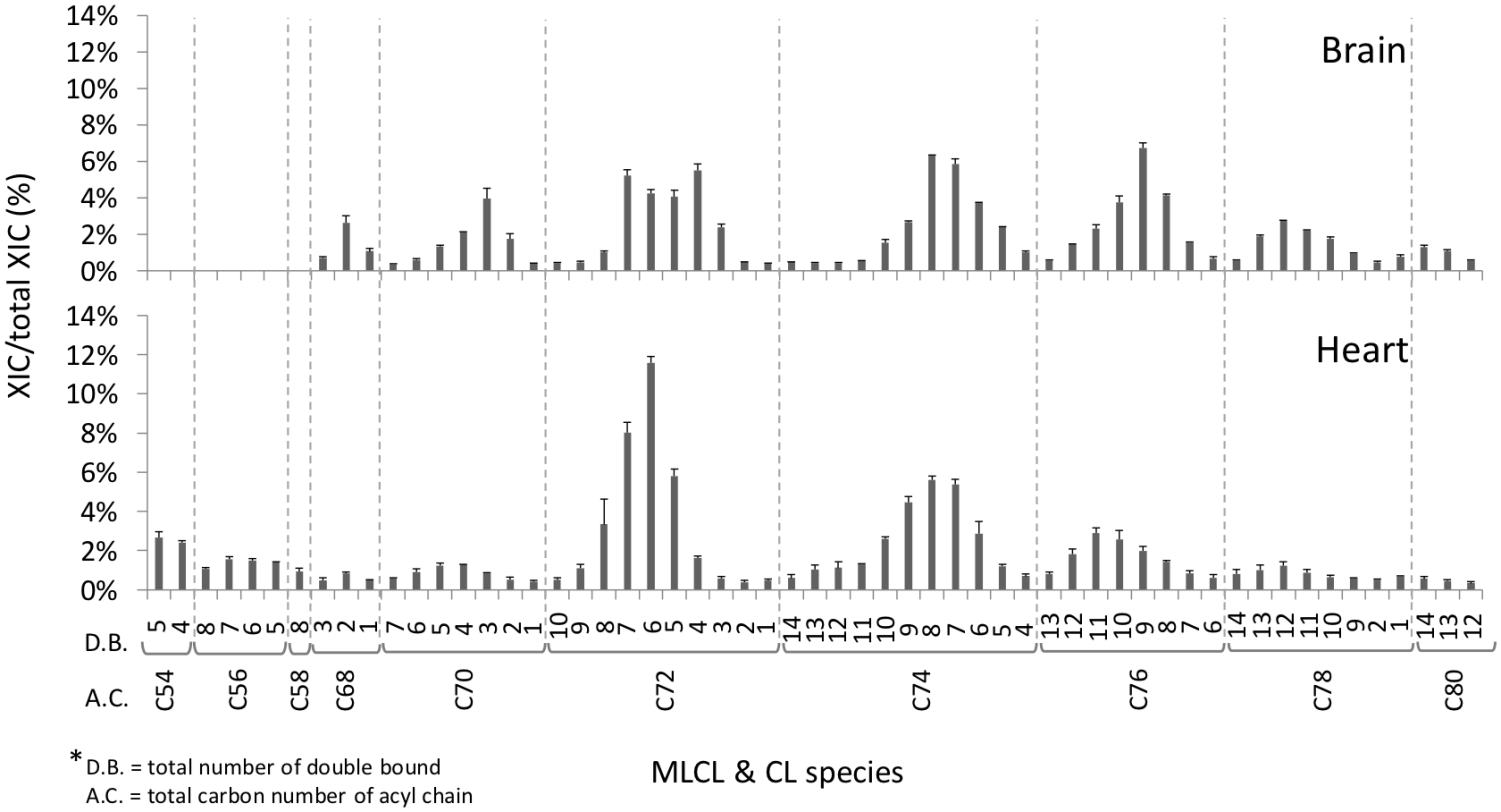
Percentage of CL and MLCL species in the brain and the heart. Adult female fish (fifteen-months old) was maintained with normal diet for four weeks. After total lipid extraction, the cardiolipin was analyzed by LC-MS. Total XIC is defined as the XIC of all detected CL and MLCL species.

The mass spectra of the brain and eye tissues are drastically different from those of the heart and muscle tissues, suggesting that these two categories of organ tissues contain distinct compositions of mitochondrial membrane phospholipids, which likely lead to different mitochondrial activities besides energy conversion, such as apoptosis signaling and production of reactive oxygen species. Quantification of CL species assists the analysis of the changes of each individual CL species. Therefore, we semi-quantified the CL and MLCL species by the relative extracted ion current (XIC) to the externally added CL (14:0)_4_ internal standard (S4 Fig). Subsequently, we compared the percentages of CL and MLCL in the brain with those in the heart (Fig 5). The C72, C74 and C76 groups are three major groups of CL in the brain. In the heart, the C72 group is the dominant group while C72:5, C72:6 and C72:7 are the top three CL species, suggesting that the heart requires these CL species to maintain the energy production of mitochondria. A higher percentage of CLs in C72, C74 and C76 groups tended to have 5 to 8 of double bonds instead of 1 to 4 double bonds. The C54, C56 and C58 groups belong to the MLCL, which cannot be detected in the brain tissue. In the heart tissue, the abundant CL species are C54, C56 and C58 MLCL and C72, C74 and C76 CL, suggesting that the hydrolysis and reacylation reaction of CL and MLCL dominantly utilizes fatty acids with 18 carbons.

**Fig 6:**
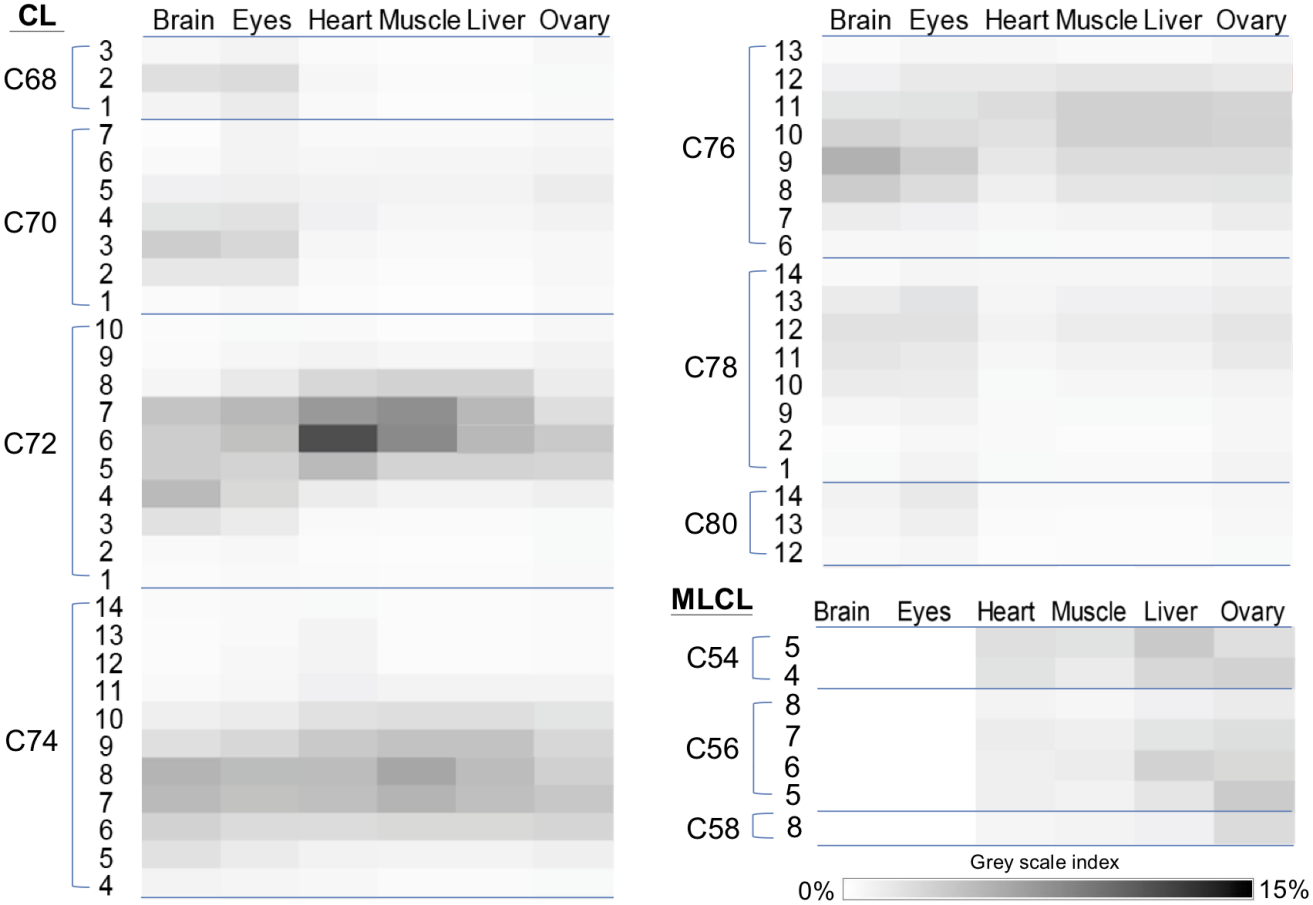
The grey scale map of the percentage of CL and MLCL species in the organs of adult fishes. The percentage of each CL and MLCL species was displayed according to the grey scale index, which is the ratio of XIC to total XIC as detected by LC-MS.

To have an overview of the CL and MLCL profiles of the organs of the adult fish, the percentages of CL and MLCL species were expressed on the gray scale map (Fig 6). C72:6 and C72:7 are the two abundant species in C72 group. This indicates that 18:1 and 18:2 are the two most abundant fatty acyl chains of CL in zebrafish. It is interesting to note that no MLCL was detected in either brain or eye tissue, and the percentages of MLCL in liver and ovary are higher than those in other tissues. These effects on CL degradation may be caused by complex reasons involving the regulation of CL remolding enzymes.

**Fig 7:**
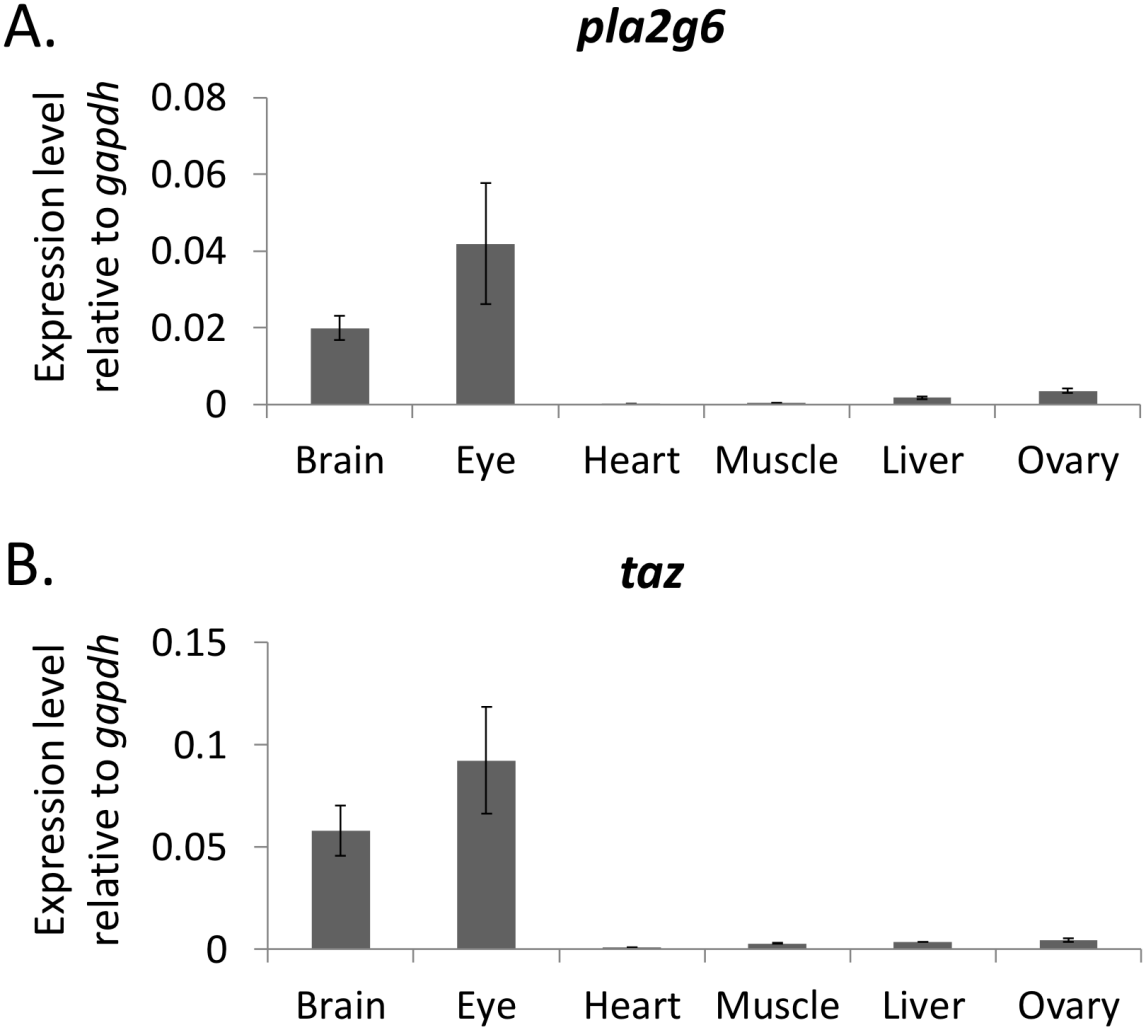
*tafazzin* and *pla2g6* gene expression in zebrafish organs. The gene expression of (A) *tafazzin* and (B) *pla2g6* are examined by RT-qPCR. The heat map representing *gapdh* is used as the reference gene.

It is possible that high percentages of MLCL are caused by either a lack of Tafazzin or high expression of iPLA_2_. Therefore, we examined the gene expression of *tafazzin* and *pla2g6* in various adult organs (Fig 7). The expression of *tafazzin* was substantially lower in tissues of heart, muscle, liver and ovary than that in the brain and eye tissues, indicating that Tafazzin is a key enzyme for regulating the MLCL concentration. The expression levels of *pla2g6* and *tafazzin* however seemed to follow the similar trend, suggesting that the hydrolysis of CL are tightly accompanied with the reacylation of MLCL in adult zebrafish organs. It remains unclear whether unidentified mechanisms of CL hydrolysis might exist in MLCL-containing organs including heart, muscle, liver and ovary.

### Chlorella diet induced changes of CL species in the adult zebrafish

**Fig 8:**
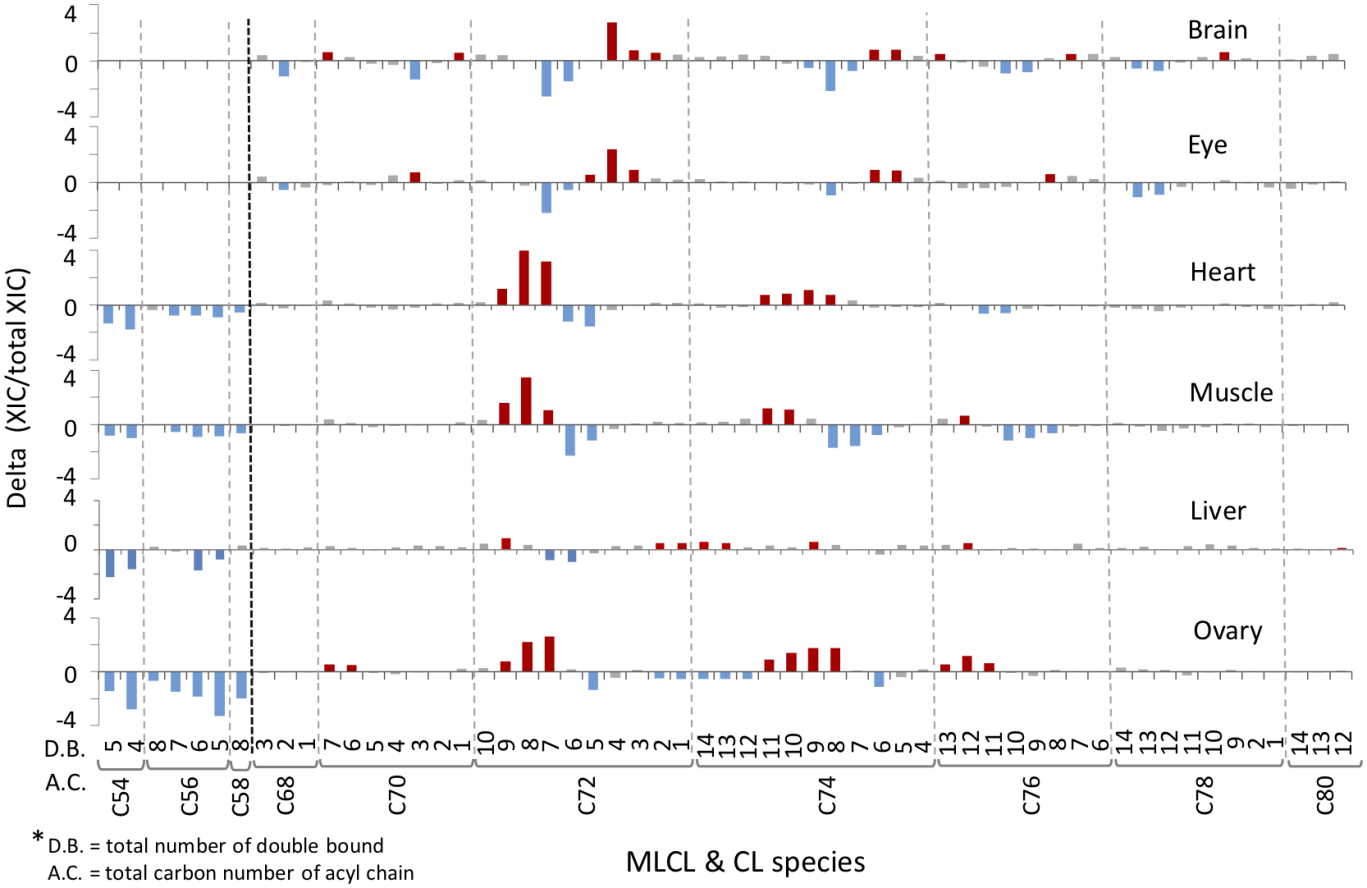
Chlorella induced changes of CL and MLCL contents in the organs of adult zebrafish. Adult female fish (fifteen-months old) was maintained with chlorella diet or control normal diet for four weeks. The lipids in each organ were extracted, and cardiolipins were analyzed by LC-MS. Delta (XIC/total XIC) indicates the percentage change for each CL or MLCL species, between the chlorella diet and the control normal diet treatments. The red and blue bars indicate increased and decreased percentages, respectively.

While the chlorella diet led to changes of CL profiles in the larval fish, we further tested whether this feeding effect could also be detected in the adult zebrafish organs. Based on the differential distribution patterns of CL in different organs, it is hypothesized that chlorella diet may cause differential effects on the CL profiles in various organs. To examine the differential effects of PUFA-abundant chlorella diet on mitochondria, the fifteen months old adult fish was maintained with chlorella diet for four weeks. Total lipids in the six isolated organs were extracted by Bligh-Dyer’s extraction method and analyzed by LC-MS (Fig 8). In the group of chlorella-supplemented diet, the C72 and C74 CL showed an increase of percentage on the species of saturated acyl chains, particularly C72:4, which is the symmetrical CL(18:1)_4_ in the brain and eye tissues. The major CL changes of heart and muscle are localized in the C72 group, which demonstrates a reverse trend of changes compared to the brain and eyes. The percentages of C72:7, C72:8 and C72:9 were elevated up to 4% to decrease the saturation of CL fatty acyl chains, suggesting an incorporation of PUFA into the heart and muscle tissues. The CL profile of the liver is relatively less influenced by the intake of chlorella. The ovary presents an increase of highly unsaturated CL and a decrease of MLCL, suggesting a reacylation of MLCL induced by the chlorella intake.

**Fig 9:**
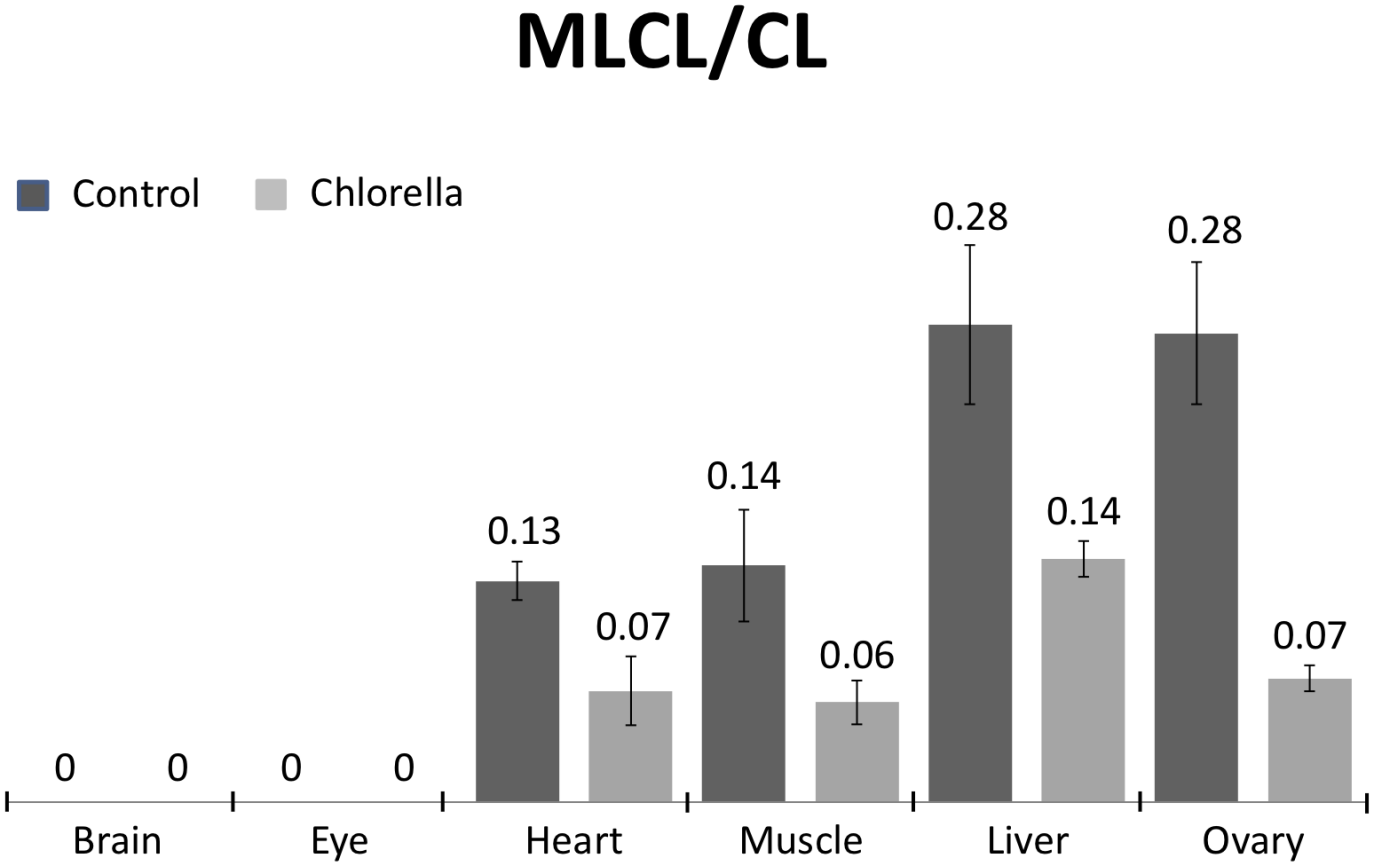
The effects of chlorella diet on MLCL and CL. Organs of the adult zebrafish were harvested after four weeks of normal and chlorella diet. The ratio of MLCL to CL is calculated by the ratio of their XIC extracted from the mass spectrum.

Reacylation of MLCL and hydrolysis of CL can change the ratio of MLCL to CL. MLCL/CL is an important index to evaluate the status of CL in the organs (Fig 9). As we have seen in the percentage changes, chlorella diet results in a 75% drop in the MLCL/CL ratio. The heart, muscle, and liver all show about 50% decrease of the MLCL/CL ratio, indicating that chlorella diet significantly increases the CL content and decreases the MLCL content. Interestingly, MLCL were shown to be absent in brain and eyes, either with standard food or with chlorella diet.

## Discussion

**Fig 10:**
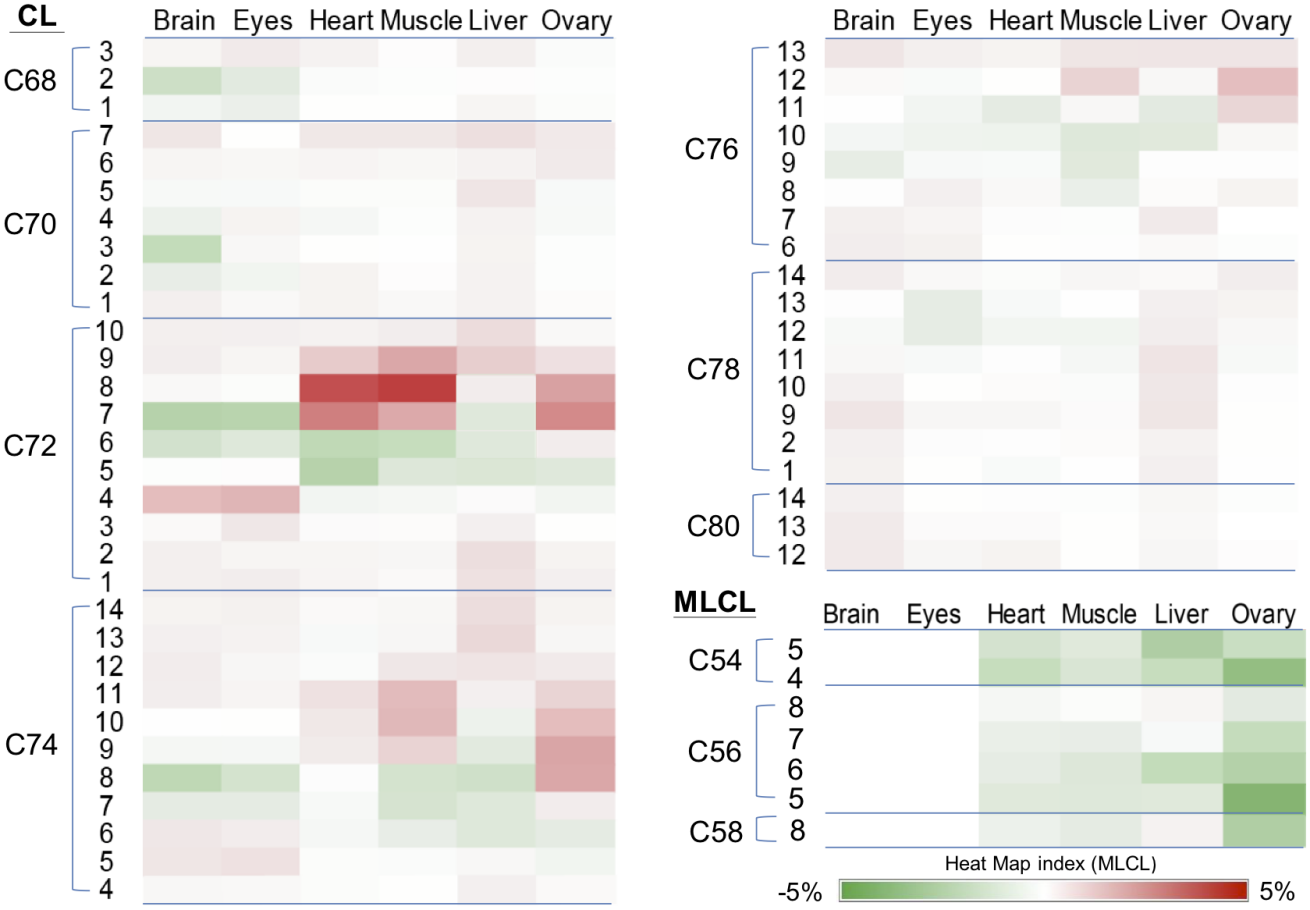
The heat map representing changes of CL and MLCL species in the organs of zebrafish. The percentage change indicates the changes between chlorella diet and the control diet, and is displayed according to the heat map index. Green indicates decreases of CL percentages; Red indicates increases of CL percentages.

Research evidences have shown that exogenous fatty acids can alter the CL contents of the palmitate-induced cardiac myocyte during apoptosis [35, 36] or the PUFA-supplemented H9c2 cardiac myoblast [37]. Similarly, CL alteration effects can also be observed in the cardiac mitochondria in rats, when DHA was supplemented in diet [38]. Chlorella has been a commercially available nutritional supplement which contains abundant diverse PUFA and long chain fatty acids. The PUFA is regarded beneficial for preventing cardiovascular diseases. In our study, chlorella diet has a significant effect on changing the CL profile, in both larval and adult zebrafish. For showing the chlorella feeding effect on the CL profile of adult organs, we illustrated the percentage changes of CL upon chlorella feeding on a heat map (Fig 10). Two significant effects can be clearly observed on this heat map. The first effect is the increase of C72:8 CL in the muscle and heart tissues. This CL (18:2)_4_ species is a symmetrical CL, which has been an important indicator of CL maturation. The symmetrical CL dominates the heart and muscle tissues of mammals. The CL species in heart and muscle also shifted from less unsaturated CL to highly unsaturated CL for at least 2 double bond differences in C72 and C74 groups. In our study using the teleostean fish model, it shows that the daily nutrition can affect the remodeling of mitochondrial phospholipids in the heart and the muscle. The second effect of chlorella diet is the decrease of MLCL in the heart, the muscle, the liver, and particularly the ovary. MLCL has been shown as an improper form of CL in the patients of Barth syndrome [39, 40]. Accumulation of MLCL caused by the tafazzin mutation can decrease the function of mitochondria [41–43]. We found that chlorella diet can drastically decrease the content of MLCL, implicating a potential beneficial effect of chlorella-supplemented diet, which is worthy of further investigation.

The average body weight of the adult female zebrafish used in the experiments was 1.8 g, and each fish was fed approximately 5 mg of food per day. The flake fish food and the chlorella contain 8.3% and 11.5% of fats respectively. Therefore, each adult fish ingested approximately 0.23 mg/g body weight/day of fats in the control group, and 0.32 mg/g body weight/day of fats in the chlorella treatment group. Among the organs of an adult fish, the heart contains 0.4 μg of CL, the liver contains 2.4 μg of CL, and two eyes contain 0.7 μg of CL. Overall, we estimate that 500 μg of fats per day, in two weeks, is enough to lead to profile changes in 0.4–2.4 μg of CL contained in various zebrafish organs.

Albeit being functionally distinct organs, the brain and eyes share similarities in the CL profile. Specifically, both organs do not contain any detectable MLCL by MS analysis, indicating that the mitochondria in these two organs only contain CL. While the chlorella diet triggered the CL remodeling in the brain and eye tissues, still no MLCL can be detected during the remodeling process. The high expression level of *tafazzin* in the brain and eyes suggests that the reacylation of MLCL to CL is active in these organs (Fig 6), thus, preventing the accumulation of MLCL. Alternatively, lyso-cardiolipin acyl-transferase (ALCAT1; LCLAT1) has been shown to be responsible for the re-acetylation of MLCL [44], and this gene also exists in zebrafish. This zebrafish homolog of ALCAT1 has been shown related to the development of hematopoietic and endothelial lineages [45]. Because the levels of tafazzin and phospholipase expressions varied among organs, LCLAT1 may provide an alternative mechanism of CL remodeling in the tafazzin-deficient tissues.

Both the heart and the muscle require large amount of energy processing and high mitochondrial activity. Cardiolipin has been recognized as an oxidative target in the heart of the aged rat [46, 47]. We found that the chlorella diet drastically increased the symmetrical CL in the heart and muscle tissues, which potentially enhance the mitochondrial activity. The induced CL remodeling may reduce the accumulation of the oxidized fatty acid in heart. The CL profile of the ovary seems to benefit most from the feeding of chlorella. Acylation of MLCL actively synthesizes new highly unsaturated CL in the ovary to decrease the total quantity of MLCL.

## Supporting information

S1 Fig Standard curve of TIC detector response versus content of cardiolipin standard. The quantification of 2.5 ng, 5 ng, 10 ng, 20 ng and 40 ng of the CL standards were triplicated and the results were evaluated by linear regression.
(DOCX)

S2 Fig Cardiolipins in the larval zebrafish after being treated with fish oil-supplemented chlorella diet. Larval fish (one-week old) was maintained with fish-oil supplemented chlorella diet for two weeks. The spectrum of cardiolipins in larval zebrafishs (A) and the percentage of CL and MLCL species (B) were analyzed by LC-MS. Total extracted ion current (XIC) is the XIC of all detected CL and MLCL.
(DOCX)

S3 Fig Mass spectrum of cardiolipins in the pig liver and the siganus muscle. The total lipids in the pig liver (A) and the siganus muscle (B) were extracted by Bligh/Dyer’s method. The cardiolipin of the lipid extract was analyzed by Ion trap MS.
(DOCX)

S4 Fig Percentage of CL and MLCL species in various organs. Adult fish (fifteen-months old) was maintained with normal diet for four weeks. After total lipid extraction, the cardiolipin was analyzed by LC-MS. Total extracted ion current (XIC) is the XIC of all detected CL and MLCL species.
(DOCX)
