# Supplementary material for: Chlorella diet alters mitochondrial cardiolipin contents differentially in organs of Danio rerio analyzed by a lipidomics approach
Source: PLoS One. 2018 Mar 1;13(3):e0193042. doi: 10.1371/journal.pone.0193042 (PMC5832209; doi:10.1371/journal.pone.0193042)
Supplement: S1 Fig — The quantification of 2.5 ng, 5 ng, 10 ng, 20 ng and 40 ng of the CL standards were triplicated and the results were evaluated by linear regression. (DOCX) [file pone.0193042.s001.docx]

**Supporting Information**

S1 Fig

**S1 Fig. Standard curve of TIC detector response versus content of cardiolipin standard.** The quantification of 2.5 ng, 5 ng, 10 ng, 20 ng and 40 ng of the CL standards were triplicated and the results were evaluated by linear regression.
