# Supplementary material for: Chlorella diet alters mitochondrial cardiolipin contents differentially in organs of Danio rerio analyzed by a lipidomics approach
Source: PLoS One. 2018 Mar 1;13(3):e0193042. doi: 10.1371/journal.pone.0193042 (PMC5832209; doi:10.1371/journal.pone.0193042)
Supplement: S2 Fig — Larval fish (one-week old) was maintained with fish-oil supplemented chlorella diet for two weeks. The spectrum of cardiolipins in larval zebrafishs (A) and the percentage of CL and MLCL species (B) were analyzed by LC-MS. Total extracted ion current (XIC) is the XIC of all detected CL and MLCL. (DOCX) [file pone.0193042.s002.docx]

**Supporting Information**

S2 Fig

**S2 Fig.** **Cardiolipins in the larval zebrafish after being treated with fish oil-supplemented chlorella diet.** Larval fish (one-week old) was maintained with fish-oil supplemented chlorella diet for two weeks. The spectrum of cardiolipins in larval zebrafishs (A) and the percentage of CL and MLCL species (B) were analyzed by LC-MS. Total extracted ion current (XIC) is the XIC of all detected CL and MLCL.
