# Supplementary material for: Chlorella diet alters mitochondrial cardiolipin contents differentially in organs of Danio rerio analyzed by a lipidomics approach
Source: PLoS One. 2018 Mar 1;13(3):e0193042. doi: 10.1371/journal.pone.0193042 (PMC5832209; doi:10.1371/journal.pone.0193042)
Supplement: S3 Fig — The total lipids in the pig liver (A) and the siganus muscle (B) were extracted by Bligh/Dyer’s method. The cardiolipin of the lipid extract was analyzed by Ion trap MS. (DOCX) [file pone.0193042.s003.docx]

**Supporting Information**

S3 Fig

**S3 Fig. Mass spectrum of cardiolipins in the pig liver and the siganus muscle.** The total lipids in the pig liver (A) and the siganus muscle (B) were extracted by Bligh/Dyer’s method. The cardiolipin of the lipid extract was analyzed by Ion trap MS.
