# Supplementary material for: Chlorella diet alters mitochondrial cardiolipin contents differentially in organs of Danio rerio analyzed by a lipidomics approach
Source: PLoS One. 2018 Mar 1;13(3):e0193042. doi: 10.1371/journal.pone.0193042 (PMC5832209; doi:10.1371/journal.pone.0193042)
Supplement: S4 Fig — Adult fish (fifteen-months old) was maintained with normal diet for four weeks. After total lipid extraction, the cardiolipin was analyzed by LC-MS. Total extracted ion current (XIC) is the XIC of all detected CL and MLCL species. (DOCX) [file pone.0193042.s004.docx]

**Supporting Information**

S4 Fig

**S4 Fig.** **Percentage of CL and MLCL species in various organs.**

Adult fish (fifteen-months old) was maintained with normal diet for four weeks. After total lipid extraction, the cardiolipin was analyzed by LC-MS. Total extracted ion current (XIC) is the XIC of all detected CL and MLCL species.
